# Influence of Occupational Background on Smoking Prevalence as a Health Inequality Among Employees of Medium- and Small-Sized Companies in Japan

**DOI:** 10.1089/pop.2019.0021

**Published:** 2020-03-11

**Authors:** Takako Fujita, Akira Babazono, Yumi Harano, Peng Jiang

**Affiliations:** ^1^ Department of Health Sciences, Faculty of Medical Sciences, Kyushu University, Fukuoka, Japan.; ^2^ Graduate School of Medical Sciences, Kyushu University, Fukuoka, Japan.; ^3^ Department of Healthcare Administration and Management, Faculty of Medical Sciences, Kyushu University, Fukuoka, Japan.

**Keywords:** smoking, occupation, health inequality, workplace, prevalence

## Abstract

Tobacco smoking is a major public health problem. In addition, the influence of socioeconomic status on health inequalities has received great attention worldwide. The authors used insurance data of beneficiaries employed in medium- and small-sized Japanese companies to investigate the influence of occupational background on smoking prevalence as a health inequality among workers in Japan. Participants were aged 35–74 years and underwent health examinations in 2015. Smoking prevalence was estimated for each occupational group according to sex, age, and income. Logistic regression analysis was used to assess the association between smoking status and occupational groups. A total of 385,945 participants were included. Overall smoking prevalence was 36.3%, higher than average in Japan. Smoking prevalence was lowest among workers in the education and learning support category; all other occupational groups had significantly high prevalence, with the highest for transport and postal services (odds ratio 2.69, 95% confidence interval 2.53–2.86). There were few differences in smoking prevalence at higher income levels among female participants, but differences were remarkably significant at lower income levels. For health inequalities related to smoking, occupational background was associated with smoking prevalence. In particular, there was high smoking prevalence in workplaces not covered by smoke-free policies. These results also demonstrated differences between the sexes; smoking prevalence among female workers with lower income levels was strongly associated with occupational background whereas there were no large differences among male workers by income. These findings suggest that the government should encourage companies to adopt smoke-free policies in the workplace.

## Introduction

Tobacco smoking is a major public health problem worldwide. Tobacco users as well as nonusers are at risk of tobacco-related illnesses. Age-adjusted smoking prevalence in 2015 was estimated at 34.1% for males and 6.4% for females globally^1^; in Japan, this prevalence was 31.4% for males and 8.3% for females.^2^ In high-income countries, smoking prevalence declined to 23.1% in 2015.^3^ Although it is a high-income country, the prevalence of smoking in Japan is higher than in other developed countries, despite an annual decline.

According to the National Health and Nutrition Survey Japan in 2017, the proportion of smokers who wanted to quit smoking was 28.9% (26.1% for male smokers and 39.0% for females).^2^ In the workplace, 39.6% of workers reported that they were exposed to secondhand smoke at work.^2^ Therefore, promoting smoke-free workplace policies is important to support smoking cessation among smokers, to provide a clean air working environment, and to protect all workers from secondhand smoke.

In Health Japan 21 (second term), basic health promotion policies in the country include the following goals by 2022: reduce the prevalence of adult smoking to 12% and the rates of exposure to secondhand smoke in restaurants, homes, and government and health care institutions to 15%, 3%, and 0%, respectively.^4^ Although the goal for workplaces is a smoke-free environment, no numerical targets have been set.^4^

The influence of socioeconomic status on health inequalities has received greater attention worldwide in recent years.^5^ The disparity in smoking prevalence according to socioeconomic status increased from 2000 to 2010, and reduction of health inequalities has become a basic direction of health promotion in Japan.^4^ Socioeconomic factors, such as education, income, and employment status, are associated with mortality and risk factors of noncommunicable diseases (NCDs), including cancer, cardiovascular diseases, and chronic respiratory diseases.^6,7^ Smoking is one of the main risk factors of NCDs and is associated with socioeconomic status; smoking prevalence is higher among individuals with lower income and lower education levels, and this prevalence differs significantly by occupation.^8–14^

With respect to the workplace, health inequalities among workers cannot only be explained by education, income, or employment status, because people of different backgrounds often work together in the same environment. Although smoke-free measures have been implemented progressively in Japan, they lag behind those of other countries.^15^ Personal socioeconomic and smoking status must be considered when assessing health inequalities; however, despite having a high socioeconomic status, a worker might experience health inequality in a workplace that permits employees to smoke anytime, owing to exposure to secondhand smoke. Because overall smoking prevalence is affected by the implementation of smoke-free laws, regulations, and policies, as well as increased tobacco prices and taxes,^16,17^ studies that focus on updating information regarding smoke-free workplace environments are needed.

Thus, this study investigated the influence of occupational background on the smoking prevalence among workers in Japan. The results could strengthen the evidence for promoting smoking cessation and smoke-free measures in the workplace, to help reduce health inequalities related to occupation.

## Methods

### Study population

The research team used data on employees in the Japan Health Insurance Association Fukuoka Branch. The Japan Health Insurance Association is an insurer for employees of medium- and small-sized companies and their family members who are younger than age 75 years. These beneficiaries represent 29.3% of the entire Japanese population.^18^ The Fukuoka Branch had 1,837,766 beneficiaries at the end of fiscal year 2015, including 1,036,636 employees.^18^

Although the Industrial Safety and Health Act requires companies to offer health examinations to prevent work-related diseases to all employees each year, the purpose of the health examination by this Act is to prevent NCDs for employees aged 35–74 years.^19^ The age 35 years includes some people who are 34 years old but who would be 35 years old at the end of the fiscal year 2015. During the examination, beneficiaries are required to complete self-reported questionnaires, which include information regarding current smoking status. The Japan Health Insurance Association administers the results of these examinations and questionnaires.

A total of 717,819 eligible employees underwent health examinations. This study included 393,930 employees who underwent a health examination in fiscal year 2015. A total of 7985 individuals were excluded because of missing information on age and smoking status.

### Outcomes

In this study, the outcome was smoking prevalence according to occupational group, considering sex, age, and income.

The research team extracted participant age on the day of the health examination from the database and divided participants into 4 age groups: 35–39, 40–49, 50–59, and 60 years and older.

The team used standardized monthly income as the income levels used to determine each individual's insurance premium including salary and allowance per month. Income level in this study was categorized into 5 groups (USD 1 = JPY 100): <JPY 150,000 (USD 1500), JPY 150,000–249,999 (USD 1500–2499), JPY 250,000–349,999 (USD 2500–3499), JPY 350,000–449,999 (USD 3500–4499), and ≥JPY 450,000 (USD 4500).

Occupation was categorized into 18 groups according to the Japan Standard Industrial Classification.^20^ According to this classification, service occupations comprised transport and postal services; scientific research, professional, and technical services; accommodation, food and beverage services; living-related and personal services and entertainment services; compound services; government services; and other services including waste disposal, automobile maintenance, machine repair, employment services, and worker dispatch services, among others.

### Statistical analysis

SQL Server 14.0 (Microsoft Corporation, Redmond, WA) was used to extract the data and Stata 15.0 (StataCorp LLC, College Station, TX) was used for the analyses.

The research team first calculated smoking prevalence for each occupational group by sex and age categories as well as income levels. Multiple logistic regression analysis was conducted to obtain odds ratios (ORs) for the associations between smoking status and occupational groups, with 95% confidence intervals for the variables sex, age category, income level, and occupational group. The same analysis also was conducted for each income level by sex to assess the influence of the workplace on socioeconomic status.

This study was approved by the Institutional Review Board of Kyushu University (Clinical Bioethics Committee of the Graduate School of Healthcare Sciences, Kyushu University), permission number 28–84. The names and residential addresses of all employees were de-identified by constructing specific databases using a workstation with no connection to any networks.

## Results

In this study, there were a total of 385,945 participants, 53.8% of whom were employees. Overall smoking prevalence was 36.3% – 46.7% for males and 18.1% for females; these rates were higher than those of the general population of Japan. Among occupational groups, the largest was transport and postal services and the smallest was government services. Additional details are provided in Table 1.

**Table 1.** Smoking Prevalence Among Study Participants in Fukuoka, Japan, 2015

	Smoking		No smoking		Total
	N	%	N	%	N
	140,059	(36.3)	245,886	(63.7)	385,945
Sex					
Male	114,741	(46.7)	131,065	(53.3)	245,806
Female	25,318	(18.1)	114,821	(81.9)	140,139
Age category					
35–39	24,931	(42.2)	34,101	(57.8)	59,032
40–49	54,853	(39.1)	85,266	(60.9)	140,119
50–59	38,971	(35.0)	72,504	(65.0)	111,475
≥ 60	21,304	(28.3)	54,015	(71.7)	75,319
Income level (JPY)					
≤ 50,000	7282	(24.0)	23,072	(76.0)	30,354
150,000–249,999	39,522	(29.9)	92,464	(70.1)	131,986
250,000–349,999	49,193	(41.6)	68,954	(58.4)	118,147
350,000–449,999	29,079	(43.8)	37,298	(56.2)	66,377
≥ 450,000	14,983	(38.3)	24,098	(61.7)	39,081
Occupational group					
Agriculture, forestry, and fisheries	1055	(34.2)	2030	(65.8)	3085
Mining and stone quarrying	327	(46.6)	374	(53.4)	701
Construction	14,098	(44.7)	17,436	(55.3)	31,534
Manufacturing	25,947	(39.2)	40,260	(60.8)	66,207
Electricity, gas, heat supply, and water	1282	(37.6)	2130	(62.4)	3412
Information and communications	2682	(33.0)	5447	(67.0)	8129
Transport and postal services	23,331	(51.1)	22,341	(48.9)	45,672
Wholesale and retail trade	25,902	(37.9)	42,528	(62.1)	68,430
Finance and insurance	1741	(28.0)	4487	(72.0)	6228
Real estate and goods rental and leasing	3713	(35.9)	6641	(64.1)	10,354
Scientific research, professional and technical services	4537	(30.6)	10,268	(69.4)	14,805
Accommodations, food and beverage services	3308	(40.5)	4869	(59.5)	8177
Living-related and personal services and entertainment services	3558	(40.3)	5260	(59.7)	8818
Education and learning support	698	(16.0)	3677	(84.0)	4375
Medical, health care, and welfare	6624	(21.0)	24,906	(79.0)	31,530
Compound services	265	(30.7)	599	(69.3)	864
Other services	11,558	(36.3)	20,256	(63.7)	31,814
Government services	1426	(15.9)	7530	(84.1)	8956
Unable to classify and unknown	8007	(24.4)	24,847	(75.6)	32,854

JPY, Japanese yen.

Table 2 shows the smoking prevalence according to occupational groups in each age category, by sex. Smoking prevalence decreased with age among male participants; the highest prevalence among female participants was among those aged 40–49 years. Table 3 shows smoking prevalence according to occupational group at each income level, by sex. The results showed greater differences between the sexes than by age category. The highest smoking prevalence was for income level JPY 250,000–349,999 (USD 2500–3499) and the lowest prevalence was for income levels less than JPY 150,000 (USD 1500). Among females, smoking prevalence declined with higher income levels. From the viewpoint of occupational groups, the results for male participants were similar to those for all participants, whereas those for female participants showed differences.

**Table 2.** Smoking Prevalence for Each Occupational Group, by Age and Sex, Fukuoka, Japan, 2015

Male smokers					
Occupational group	Age category				
	35–39	40–49	50–59	≥60	Total
	%				
Agriculture, forestry, and fisheries	52.3	50.3	47.2	32.5	43.8
Mining and stone quarrying	66.1	59.3	55.1	32.4	52.3
Construction	60.4	56.3	50.2	36.7	50.7
Manufacturing	54.0	49.1	45.6	36.3	46.8
Electricity, gas, heat supply, and water	53.8	49.3	46.3	27.6	42.5
Information and communications	37.5	38.4	39.4	33.2	38.1
Transport and postal services	62.6	56.6	54.0	46.3	53.8
Wholesale and retail trade	54.2	50.9	45.2	35.0	47.8
Finance and insurance	43.5	43.3	39.7	31.3	40.2
Real estate and goods rental and leasing	52.9	48.2	41.6	31.7	44.0
Scientific research, professional and technical services	45.8	41.5	37.1	27.3	38.3
Accommodations, food and beverage services	56.5	55.4	47.7	36.7	51.4
Living-related and personal services and entertainment services	60.9	53.6	46.5	34.4	50.0
Education and learning support	42.3	32.8	31.5	22.4	31.5
Medical, health care, and welfare	45.3	40.0	34.4	29.5	37.9
Other services	54.8	50.3	47.4	32.9	45.1
Government services	34.6	41.5	39.8	30.4	32.0
Total	53.8	50.2	46.4	35.7	46.7

Female smokers					
Occupational group	Age category				
	35–39	40–49	50–59	≥60	Total
	%				
Construction	19.5	18.0	15.3	8.6	15.6
Manufacturing	21.3	22.7	19.3	10.5	19.0
Information and communications	14.1	17.8	15.1	14.3	16.1
Transport and postal services	25.5	28.3	24.4	19.9	25.5
Wholesale and retail trade	21.1	23.6	21.0	13.9	20.8
Real estate and goods rental and leasing	20.6	20.8	18.7	8.0	18.4
Scientific research, professional and technical services	13.9	14.4	14.4	9.4	13.8
Accommodations, food and beverage services	24.9	28.4	28.1	18.6	25.5
Living-related and personal services and entertainment services	31.4	32.4	28.5	16.5	27.8
Medical, health care, and welfare	18.2	19.5	17.7	10.9	17.5
Other services	18.6	19.4	17.8	16.6	18.2
Government services	9.5	7.5	7.2	4.1	6.9
Total	19.3	20.3	18.2	11.8	18.1

Only those occupational groups with a total of more than 300 participants are shown.

**Table 3.** Smoking Prevalence for Each Occupational Group by Income Level and Sex, Fukuoka, Japan, 2015

Male smokers						
Occupational group	Income levels (JPY)					
	150,000 ≥	150,000–249,999	250,000–349,999	350,000–449,999	≥ 450,000	Total
	%					
Agriculture, forestry, and fisheries	39.3	40.8	50.0	44.9	43.2	43.8
Mining and stone quarrying	50.0	46.8	54.8	53.4	48.5	52.3
Construction	34.2	49.0	53.7	51.8	46.3	50.7
Manufacturing	34.5	45.0	49.1	47.6	43.3	46.8
Electricity, gas, heat supply, and water	30.0	34.6	45.8	47.5	38.2	42.5
Information and communications	35.0	32.6	37.3	39.1	40.7	38.1
Transport and postal services	52.8	51.6	56.2	54.6	46.9	53.8
Wholesale and retail trade	39.5	44.8	51.0	48.7	43.7	47.8
Finance and insurance	36.2	38.8	41.0	42.0	39.2	40.2
Real estate and goods rental and leasing	27.8	41.4	48.5	46.6	40.8	44.0
Scientific research, professional and technical services	29.9	34.9	41.2	40.6	35.4	38.3
Accommodations, food and beverage services	38.0	48.3	55.4	52.2	44.4	51.4
Living-related and personal services and entertainment services	32.5	47.1	55.0	50.4	46.7	50.0
Education and learning support	34.1	28.2	33.2	31.1	31.1	31.5
Medical, health care, and welfare	29.2	44.2	42.1	37.2	25.1	37.9
Other services	32.8	41.8	49.4	48.7	42.2	45.1
Government services	34.3	32.8	27.8	31.0	22.6	32.0
Total	37.4	44.7	50.0	48.2	41.3	46.7

Female smokers						
Occupational group	Income levels (JPY)					
	150,000 ≥	150,000–249,999	250,000–349,999	350,000–449,999	≥ 450,000	Total
	%					
Construction	17.1	14.9	17.2	14.0	14.9	15.6
Manufacturing	19.4	19.5	17.4	16.6	10.6	19.0
Information and communications	14.7	17.2	15.1	14.5	20.3	16.1
Transport and postal services	26.5	26.2	26.4	21.5	6.9	25.5
Wholesale and retail trade	22.2	21.6	18.2	17.4	13.7	20.8
Real estate and goods rental and leasing	15.9	20.2	18.0	15.9	11.2	18.4
Scientific research, professional and technical services	16.2	13.3	13.7	13.2	16.3	13.8
Accommodations, food and beverage services	17.5	28.5	24.0	31.9	18.6	25.5
Living-related and personal services and entertainment services	21.5	29.7	27.8	26.5	20.3	27.8
Medical, health care, and welfare	14.4	19.6	16.6	13.7	8.0	17.5
Other services	20.3	18.8	15.4	11.4	14.1	18.2
Government services	9.7	6.3	5.0	4.3	0.0	6.9
Total	18.6	19.1	16.9	14.5	11.3	18.1

Only those occupational groups with a total of more than 300 participants are shown.

JPY, Japanese yen.

The results of logistic regression analysis for smoking prevalence are shown in Table 4. The references were those variables with the lowest smoking prevalence: females, younger than 40 years of age, income level <JPY 150,000 (USD 1500), and government services occupation.

**Table 4.** Results of Logistic Regression, Fukuoka, Japan, 2015

	Univariate			Multivariate		
	ORs	95%CI		ORs	95%CI	
Sex						
Male	3.97	3.91	4.03	3.87	3.80	3.95
Age category						
40–49	0.88	0.86	0.90	0.91	0.89	0.93
50–59	0.74	0.72	0.75	0.79	0.77	0.80
≥ 60	0.54	0.53	0.55	0.49	0.47	0.50
Income level (JPY)						
150,000–249,999	1.35	1.32	1.39	1.05	1.01	1.08
250,000–349,999	2.26	2.20	2.33	0.99	0.96	1.03
350,000–449,999	2.47	2.40	2.55	0.92	0.89	0.95
≥ 450,000	1.97	1.91	2.04	0.78	0.75	0.81
Occupational group						
Agriculture, forestry, and fisheries	2.74	2.50	3.01	1.94	1.76	2.13
Mining and stone quarrying	4.62	3.94	5.41	2.41	2.05	2.84
Construction	4.27	4.02	4.54	2.28	2.14	2.43
Manufacturing	3.40	3.21	3.61	1.93	1.82	2.05
Electricity, gas, heat supply, and water	3.18	2.91	3.48	1.70	1.55	1.86
Information and communications	2.60	2.42	2.80	1.31	1.22	1.42
Transport and postal services	5.51	5.20	5.85	2.69	2.53	2.86
Wholesale and retail trade	3.22	3.03	3.41	2.03	1.91	2.16
Finance and insurance	2.05	1.89	2.22	1.39	1.28	1.51
Real estate and goods rental and leasing	2.95	2.75	3.16	1.79	1.66	1.92
Scientific research, professional and technical services	2.33	2.18	2.49	1.39	1.29	1.49
Accommodations, food and beverage services	3.59	3.34	3.85	2.43	2.25	2.61
Living-related and personal services and entertainment services	3.57	3.33	3.83	2.50	2.33	2.69
Education and learning support	1.00	0.91	1.11	0.80	0.72	0.88
Medical, health care, and welfare	1.57	1.47	1.66	1.55	1.46	1.65
Compound services	2.34	2.00	2.73	1.62	1.38	1.90
Other services	3.01	2.83	3.20	1.89	1.78	2.02
Unable to classify and unknown	1.17	1.03	1.33	0.81	0.71	0.93

CI, confidence interval; JPY, Japanese yen; OR, odds ratio.

ORs of smoking prevalence tended to decline significantly with age in both univariate and multivariate analyses. In univariate analysis, all income levels showed significantly high ORs for smoking prevalence. After adjustment of variables, ORs for income level JPY 150,000–249,999 (USD 1500–2499) were significantly high; those for income level <JPY 350,000 (USD 3500) were significantly low. The results of multivariate analysis showed that the education and learning support occupational group had the lowest ORs whereas all other groups had significantly high ORs, with the highest for transport and postal services.

Table 5 shows results of the same analysis conducted for income level by occupational groups adjusted for sex and age, and Table 6 shows these results according to sex. Mining and stone quarrying, construction, living-related and personal services, entertainment services, and transport and postal services all had significantly high ORs for smoking prevalence at all income levels. Lower income levels showed significant differences by occupational group. In particular, female workers mostly had no significant differences in smoking prevalence at higher income levels, but these differences were remarkably significant at lower levels of income.

**Table 5.** Results of Logistic Regression Analysis Adjusted for Sex and Age at Each Income Level, Fukuoka, Japan, 2015

	JPY 150,000≥			JPY 150,000–249,999			JPY 250,000–349,999			JPY 350,000–449,999			≥ JPY 450,000		
	OR	95%CI		OR	95%CI		OR	95%CI		OR	95%CI		OR	95%CI	
Agriculture, forestry, and fisheries	1.95	1.59	2.40	1.90	1.63	2.21	2.08	1.63	2.67	1.44	0.88	2.37	2.18	0.89	5.34
Mining and stone quarrying	4.55	1.43	14.51	1.75	1.26	2.44	2.62	1.95	3.50	2.11	1.21	3.66	2.98	1.14	7.82
Construction	1.73	1.43	2.09	2.03	1.87	2.22	2.46	2.06	2.93	1.89	1.20	2.96	2.43	1.05	5.64
Manufacturing	1.78	1.52	2.08	1.92	1.78	2.07	1.96	1.65	2.33	1.55	0.99	2.43	2.11	0.91	4.89
Electricity, gas, heat supply, and water	1.30	0.78	2.16	1.41	1.20	1.65	1.80	1.47	2.22	1.61	1.01	2.58	1.79	0.75	4.27
Information and communications	1.32	0.94	1.87	1.21	1.05	1.40	1.18	0.98	1.43	1.04	0.66	1.64	1.79	0.77	4.16
Transport and postal services	3.64	3.07	4.31	2.75	2.54	2.97	2.76	2.32	3.29	2.06	1.32	3.23	2.36	1.02	5.47
Wholesale and retail trade	2.09	1.79	2.43	2.05	1.90	2.21	2.05	1.72	2.45	1.59	1.02	2.49	2.10	0.91	4.86
Finance and insurance	1.11	0.84	1.47	1.24	1.07	1.42	1.32	1.06	1.64	1.13	0.72	1.80	1.75	0.75	4.07
Real estate and goods rental and leasing	1.35	1.08	1.68	1.85	1.66	2.06	1.91	1.59	2.31	1.47	0.93	2.31	1.83	0.79	4.26
Scientific research, professional and technical services	1.27	1.02	1.60	1.22	1.10	1.36	1.44	1.20	1.73	1.17	0.74	1.84	1.54	0.67	3.58
Accommodations, food and beverage services	1.70	1.36	2.12	2.75	2.48	3.05	2.49	2.06	3.02	1.88	1.19	2.99	2.18	0.92	5.13
Living-related and personal services and entertainment services	1.88	1.51	2.34	2.82	2.55	3.11	2.61	2.15	3.16	1.76	1.11	2.79	2.39	1.02	5.59
Education and learning support	0.70	0.52	0.93	0.63	0.53	0.74	0.92	0.73	1.15	0.77	0.47	1.26	1.23	0.50	3.01
Medical, health care, and welfare	1.27	1.07	1.51	1.89	1.75	2.04	1.61	1.35	1.92	1.08	0.69	1.70	0.96	0.41	2.23
Compound services	1.31	0.77	2.22	1.72	1.32	2.24	1.73	1.27	2.36	1.12	0.63	2.02	2.49	0.83	7.43
Other services	1.89	1.60	2.23	1.83	1.69	1.98	1.99	1.67	2.38	1.61	1.02	2.52	2.09	0.90	4.85

CI, confidence interval; JPY, Japanese yen; OR, odds ratio.

**Table 6.** Results of Logistic Regression Analysis Adjusted for Sex and Age at Each Income Level by Sex, Fukuoka, Japan, 2015

Male	JPY 150,000≥			JPY 150,000–249,999			JPY 250,000–349,999			JPY 350,000–449,999			≥ JPY 450,000		
	OR	95%CI		OR	95%CI		OR	95%CI		OR	95%CI		OR	95%CI	
Agriculture, forestry, and fisheries	1.14	0.82	1.59	1.14	0.94	1.37	1.71	1.31	2.24	1.31	0.79	2.20	2.10	0.85	5.22
Mining and stone quarrying	1.71	0.10	28.02	1.44	0.97	2.12	2.20	1.61	3.00	1.94	1.10	3.42	2.66	1.00	7.06
Construction	0.96	0.72	1.28	1.58	1.42	1.76	2.06	1.69	2.51	1.74	1.09	2.78	2.31	0.99	5.40
Manufacturing	0.91	0.69	1.20	1.24	1.13	1.37	1.62	1.33	1.97	1.42	0.89	2.27	2.02	0.86	4.71
Electricity, gas, heat supply, and water	0.88	0.43	1.80	0.99	0.82	1.18	1.48	1.18	1.86	1.49	0.92	2.44	1.70	0.71	4.08
Information and communications	0.87	0.54	1.40	0.66	0.55	0.80	0.94	0.76	1.16	0.95	0.59	1.52	1.67	0.71	3.93
Transport and postal services	2.07	1.60	2.68	1.81	1.65	1.99	2.26	1.86	2.76	1.89	1.18	3.02	2.28	0.97	5.33
Wholesale and retail trade	1.07	0.82	1.40	1.25	1.12	1.38	1.70	1.40	2.07	1.45	0.91	2.32	2.00	0.86	4.67
Finance and insurance	0.85	0.56	1.30	1.03	0.84	1.26	1.18	0.92	1.52	1.12	0.69	1.82	1.67	0.71	3.93
Real estate and goods rental and leasing	0.70	0.51	0.96	1.15	0.99	1.33	1.57	1.27	1.94	1.34	0.83	2.17	1.75	0.75	4.11
Scientific research, professional and technical services	0.68	0.49	0.95	0.83	0.72	0.96	1.20	0.97	1.47	1.08	0.67	1.73	1.44	0.62	3.38
Accommodations, food and beverage services	0.96	0.64	1.43	1.37	1.17	1.59	2.00	1.61	2.47	1.65	1.02	2.67	2.02	0.85	4.80
Living-related and personal services and entertainment services	0.80	0.55	1.18	1.38	1.19	1.59	1.99	1.60	2.47	1.56	0.97	2.53	2.22	0.94	5.25
Education and learning support	0.86	0.52	1.43	0.63	0.49	0.80	0.84	0.65	1.08	0.73	0.44	1.23	1.22	0.49	3.03
Medical, health care, and welfare	0.70	0.52	0.95	1.12	1.01	1.25	1.17	0.95	1.43	0.92	0.58	1.48	0.89	0.38	2.09
Other services	0.90	0.68	1.18	1.18	1.07	1.31	1.68	1.38	2.05	1.51	0.94	2.42	1.98	0.85	4.64

Female	JPY 150,000≥			JPY 150,000–249,999			JPY 250,000–349,999			JPY 350,000–449,999			≥ JPY 450,000		
	OR	95%CI		OR	95%CI		OR	95%CI		OR	95%CI		OR	95%CI	
Construction	2.32	1.76	3.07	2.52	2.15	2.97	3.82	2.40	6.10	3.75	0.49	28.65	1.15	0.69	1.91
Manufacturing	2.55	2.08	3.12	3.56	3.11	4.08	3.79	2.41	5.96	4.27	0.57	32.24	0.75	0.44	1.28
Information and communications	1.54	0.91	2.61	2.83	2.27	3.54	3.05	1.87	4.97	3.33	0.43	25.64	1.40	0.70	2.77
Transport and postal services	3.87	2.94	5.08	5.09	4.36	5.95	6.33	3.99	10.04	5.69	0.75	43.48	0.43	0.19	0.98
Wholesale and retail trade	2.97	2.44	3.62	3.99	3.49	4.56	3.97	2.54	6.20	4.58	0.61	34.29	0.96	0.62	1.50
Real estate and goods rental and leasing	2.05	1.49	2.84	3.57	3.00	4.25	3.93	2.44	6.33	4.05	0.53	31.01	0.75	0.40	1.42
Scientific research, professional and technical services	1.93	1.39	2.69	2.16	1.81	2.57	2.79	1.76	4.45	3.15	0.41	23.92	1.14	0.65	2.02
Accommodations, food and beverage services	2.41	1.83	3.18	6.05	5.16	7.10	5.67	3.52	9.15	10.39	1.33	81.15	1.43	0.70	2.93
Living-related and personal services and entertainment services	2.98	2.27	3.91	6.30	5.40	7.35	6.97	4.35	11.19	7.48	0.96	58.38	1.57	0.77	3.17
Medical, health care, and welfare	1.77	1.43	2.21	3.57	3.14	4.07	3.56	2.29	5.54	3.35	0.45	24.96	0.51	0.33	0.78
Other services	2.95	2.37	3.67	3.46	3.00	3.99	3.29	2.07	5.20	2.78	0.37	21.16	-		

CI, confidence interval; JPY, Japanese yen; OR, odds ratio.

## Discussion

The present study focused on smoking prevalence among occupational groups in Japan and revealed 3 important findings. Smoking prevalence among employees of medium- and small-sized companies was higher than that of the general Japanese population. As for health inequalities related to smoking, the prevalence of occupational smoking was significantly different after considering other variables. In addition, differences between the sexes were evident; female smoking prevalence was strongly associated with occupational groups that have lower income levels. Each of the these 3 points will be addressed.

First, study participants were insured employees of medium- and small-sized companies, and smoking prevalence in this population was higher in both male and female workers than the average across Japan. A previous study in Japan showed that smoking prevalence was nonsignificantly higher in medium- and small-sized companies than in large ones.^21^ These findings suggest that company size might be associated with prevalence of smoking.

Second, occupational group was a major factor associated with smoking prevalence, after considering age and income. Smoking prevalence varied greatly by occupation. Some occupational groups are covered by smoke-free measures, such as government services, education and learning support, and medical, health care, and welfare. Workers in these occupations had a low prevalence of smoking.

The highest smoking prevalence was seen among workers in transport and postal services, mining and stone quarrying, and construction. Construction workers have been shown to have particularly high smoking prevalence,^14,22^ as seen in the present study. Workers in the aforementioned occupational groups might smoke or may be readily exposed to secondhand smoke.

These 3 occupational groups showed highly significant ORs; however, other occupations with similarly high ORs included living-related and personal services and entertainment services; accommodations, food and beverage services, and wholesale and retail trade. These occupational groups involve mostly small-sized companies and interpersonal services, which may include serving customers who smoke. Therefore, in these occupational groups, workers themselves may smoke in the workplace, or they may be exposed to secondhand smoke from their customers. In such cases, it may be difficult to prohibit customers from smoking or to implement voluntary smoke-free workplace policies. Therefore, workers in these occupations need to be protected by laws much more so than those in other occupational groups.

Fujishiro et al suggested that occupation-specific characteristics might be related to environmental tobacco smoke exposure, independent of income and education; blue-collar workers have been found to be significantly more likely to be exposed to tobacco smoke than other occupational groups.^23^ In this study, smoking prevalence was significantly high at all income levels among mining and stone quarrying, construction, living-related and personal services, entertainment services, and transport and postal services. In addition, most occupational groups showed significant associations with smoking at lower income levels. These results suggest the existence of large health inequalities in smoking according to workplace.

Third, present study results demonstrated that differences between the sexes exist for smoking prevalence among workers. This study found that the lowest smoking prevalence was among male workers with low income levels, and the highest prevalence was among female workers with lower income levels. These results differ from those of an investigation in 2014, which suggested that high-income groups had significantly lower smoking prevalence.^24^

With respect to age groups, smoking prevalence differs between low-income and high-income countries,^16^ especially among women.^25^ A 2015 investigation in Japan reported that the highest prevalence of current smokers was among men in their 30s (41.9%), and was 11.7% among women in their 40s; smoking prevalence tended to decline in older age groups.^26^ These findings are similar to present study results.

Some studies have reported that health inequalities have emerged for smoking among young females in southern Europe^27^ and for diabetes among elderly adults in Japan.^28^ Present study findings also suggest that smoking prevalence among female participants with lower income levels was affected largely by occupation, resulting in health inequalities.

Because employed people spend most of their time in the workplace, the association between workplace environment and health is strong. Workers of different sexes, income levels, and occupations spend time together at work; therefore, it is important to assess smoking prevalence in the workplace. A high prevalence of smoking has been reported in workplaces without smoke-free policies.^14^ In work environments with a high prevalence of smoking, the risk of exposure to secondhand smoke at work is high. In addition, smokers wishing to quit might find it difficult to stop smoking under such conditions.

Although Japan ratified the World Health Organization Framework Convention on Tobacco Control in 2004, implementation of smoke-free measures in Japan lags behind those in other developed countries.^15^ The Law for Partial Amendment to the Health Promotion Act was enacted in 2018 and will be implemented in 2020. However, this law permits smoking in restaurants and establishes smoking areas in health care institutions, schools, companies, and railway stations. Therefore, workers in related occupations still will be able to smoke frequently or will continue to be exposed to secondhand smoke.

A 2017 investigation in Japan revealed the exposure to secondhand smoke among nonsmokers by location: 42.4% in restaurants, 37.3% at locations related to leisure activities, 31.7% on the street, and 30.1% in the workplace.^2^ People who work in these locations must remain in an environment where they can easily smoke or be exposed to secondhand smoke; smoking cessation also would be hampered in such conditions.

The government of Japan has been strategically promoting health and productivity management as an approach to considering the health management of employees from a corporate perspective^29^; however, individuals who work in an environment that is not covered by smoke-free laws or voluntary policies will not be protected. In other developed countries, the reported proportion of indoor workers covered by such policies has increased overall, such as among white-collar and blue-collar workers, as well as service workers.^30^ Even so, comprehensive smoke-free policies in very small workplaces are not very common^31^; therefore, much stronger laws are needed to create working environments that are indeed smoke free.

Implementation of policies to establish completely smoke-free workplaces are associated with a reduction in smoking prevalence. Tobacco tax increases would affect all tobacco users, including those in low socioeconomic groups, which would lead to a decline in overall smoking prevalence.^32,33^ The Japanese government has initiated various approaches to reduce health inequalities related to socioeconomic factors. However, the results of a study in Japan among workers in 2013 and employers in 2014 showed that more than 60% of participants did not know any of the smoke-free measures promoted by the government for implementation in the workplace, and that proportion increased in smaller workplaces.^34^

In the present study, occupational groups with a high proportion of workers who were unaware of smoke-free measures and had more than 40% smoking prevalence were living-related and personal services, entertainment services, transportation and postal services, and construction. Wada et al suggested that it is difficult for workers in transport industries to stop smoking.^35^ There is strong evidence for the beneficial effects of group therapy, individual counseling, pharmacological treatments, and multiple interventions in smoking cessation.^36^ The present study suggests that the government should encourage companies with a high prevalence of smoking to adopt smoke-free measures and smoking cessation policies in the workplace.

Participants in the present study were insured employees of medium- and small-sized companies; employees of large companies and self-employed or unemployed people were not included. In addition, participants' job titles were not considered. The reported smoking prevalence among unemployed people is higher than that of employed workers.^13,37^ In Japan, people who are unemployed for more than 1 year report a significantly high prevalence of smoking.^17^ This study focused on the smoking environment at workplaces using employee data; therefore, clarifying the smoking prevalence among unemployed people, as well as how to support smoking cessation in the workplace, remain to be addressed in the future.

The research team used standardized monthly income of individuals as income levels in this study. Because the insurance has only data on standardized monthly income related to income, the team was not able to define household size or other assets. The team might consider more precise results if that information was available to them.

Smoking and educational status have been suggested to be associated with smoking.^10,38,39^ The research team was unable to take this into consideration because study data did not include educational status. Individuals with both lower and higher levels of education are likely to work together in the same environment. Previous research has shown that lower education levels are associated with higher levels of nicotine dependence, low self-efficacy in quitting smoking, or no intention to quit smoking^40^; thus, education levels must be considered to support smoking cessation efforts in the workplace.

Finally, the team could not assess each workplace smoke-free policy because employers are not required to report these. Further research is necessary to clarify the relationship between the degree of implementation of smoke-free measures and the prevalence of smoking in the workplace in Japan.

## Conclusion

The present study focused on smoking prevalence among occupational groups in Japan. Smoking prevalence among employees of medium- and small-sized companies was higher than that of the general Japanese population. For health inequalities related to smoking, occupational background was associated with smoking prevalence. In particular, there was a high prevalence of smoking in workplaces not covered by smoke-free policies, such as in transport and postal services, mining and stone quarrying, and construction. Results showed differences between the sexes; smoking prevalence among female workers with lower income levels was strongly associated with occupational background whereas there were no large differences among male workers by income. These findings suggest that the Japanese government should encourage companies to adopt smoke-free policies in the workplace.
